# Maternal Impressions

**Published:** 1882-04

**Authors:** 


### MATERNAL IMPRESSIONS.

One's unbelief regarding the influence of maternal impressions is almost staggered by a report (*Amer. Jour. Med. Sci.*, Jan. 1881) of a most curious case under care of Dr. William Hunt, of Pennsylvania Hospital. A woman eight and a half months pregnant was admitted for extensive burns of arms, legs, and back, of which she died in three days, having given birth to a stillborn child the day following the injury. The fœtal heart-sounds were heard within six hours of birth. The important fact to record is that the child was apparently burnt and blistered in extent and places almost exactly corresponding to the injuries of the mother. The blisters on the child were standing out fresh and full as though recently formed, and in places the derm was also involved, as in the mother. Various explanations—pemphigus, syphilis, maceration, etc. —were considered untenable.

### STRYCHNIA POISONING IN A DOG CURED BY CHLORAL HYDRATE.

Dr. W. P. Gibbons, of Alameda, writes, in a note to the editors : " A large St. Bernard dog was recently poisoned by strychnia. When seen about six hours afterward, his posterior extremities were so affected with clonic spasms that he could neither walk nor stand ; every indication pointed to a fatal result. Two drachms of chloral hydrate were dissolved in an ounce of water, and I ordered one fourth to be given at a dose, repeated every hour. The first dose gave marked relief in a few minutes, and the animal recovered."—*Pacific Med. and Surg. Journal.*

### EXCISION OF THE KNEE.

1. Excision of the knee should not be looked upon as a last resource, but should be undertaken, if possible, before any profound organic changes take place.

2. Expectant treatment, to be efficient, must be undertaken at an early stage of the disease, and extend over a period of at least two years.

3. No better result than ankylosis can be looked for by this method.

4. In a patient with a predisposition to secondary tuberculous developments, the possibility of the recurrence of the disease after expectant treatment must be borne in mind.

5. In cases attended with prolonged suppuration, the chances of the occurrence of visceral, especially renal disease, must not be lost sight of.

6. Where the skin is unbroken, the disease limited, an efficient method of fixation applied, and a rigid system of antiseptic dressing of the wound adopted, primary union may, in the majority of cases, be anticipated.

7. When these latter conditions are fulfilled, excision of the knee-joint cannot be longer regarded as the formidable procedure it was formerly held to be.

8. The alleged unfavorable results of excision of the knee-joint in early life are opposed to more extended clinical experience.—*Stokes, British Med. Journal.—Detr. Lancet.*

